# Antioxidant and kidney protection; differential impacts of single and whole natural antioxidants

**DOI:** 10.12861/jrip.2014.14

**Published:** 2013-11-03

**Authors:** Mohamad Reza Tamadon, Azar Baradaran, Mahmoud Rafieian-Kopaei

**Affiliations:** ^1^Department of Internal Medicine, Semnan University of Medical Sciences, Semnan, Iran; ^2^Department of Clinical Pathology, Isfahan University of Medical Sciences, Isfahan, Iran; ^3^Medical Plants Research Center, Shahrekord University of Medical Sciences, Shahrekord, Iran

**Keywords:** Antioxidants, Oxidative stress, Herbal medicines, Polyphenols

**Implication for health policy/practice/research/medical education:**

Oxidative stress is caused by an imbalance in production of reactive oxygen and the biological ability to detoxify the reactive intermediates or repair the resulting damage. Herbal medicines commonly fight these complications with their antioxidant properties. However, it should be noted that herbal drugs extracts are abundant sources of polyphenols and these compounds are unstable and might be subjected to polymerization. Thus, it is essential to check that the observed biological properties are not due to polymerization of phenolic compounds.

Oxidative stress is caused by an imbalance in production of reactive oxygen and the biological ability to detoxify the reactive intermediates or repair the resulting damage (1). Herbal medicines commonly fight these complications with their antioxidant properties (1,2). Various preclinical and epidemiological investigations have found an inverse correlation between the intake of vegetables, fruits and/or grains which generally are rich in antioxidants and induction of diseases in humans. Antioxidants are substances that remove, delay or prevent oxidative damage to target molecules (1-3). Hence, an antioxidant may act to control the quantity of free radicals to neutralize oxidative damage (1-3). In fact there is a lot of evidence on protective and curative properties of herbal medicines on various complications. Some of these properties consist of anti-cancer, anti-diabetic, antimicrobial, immunomodulatory, amnesia, anti-atherosclerosis, kidney protection or even renoprevention effects (1-4). Herbal medicines are reliable sources of polyphenols with antioxidant activities and their beneficial properties have been attributed to their antioxidant mechanisms (1,2). There has also been a linear association between oxygen radical absorbance capacity values and total phenolic contents in several medicinal plants. For example, the equivalent antioxidant capacity values of several forms of garlic extracts were correlated well with their total phenolic, flavonoid and flavonol contents (3-5). Although medicinal plants effects in prevention and treatment of disorders have been widely attributed to their antioxidants activities, however, there is increasing evidence pointing to their pro-oxidant hazardous effects, too. This property of medicinal plants is mostly contributed to their formulae preparation (1-5). Some of polyphenols have shown to be readily oxidized in preparation of beverages like green tea. The prescription formulae preparation in traditional medicine usually involves a long decoction process with water and medicinal plants for several hours. Polyphenols in the medicinal plants in this process or storage may be oxidized. This oxidation critically reduces the beneficial properties of herbal medicine products (2-5). Pro-oxidant, as a chemical substance, induces oxidative stress, either by generating reactive oxygen species (ROS) or by inhibiting antioxidant systems. The oxidative stress induced by this substance can induce injury to the cells and tissues (1-6). Polyphenols in herbal medicines can act as either antioxidants or pro-oxidants, depending on situations. Transition metals like iron, copper and manganese are present in most cases of pro-oxidant activities of substances (1-6). Furthermore, polyphenols are usually present in medicinal plants in high concentration and are metabolized by phase I (cytochromes P450) and phase II (sulfotransferases, glucuronyl transferases and glutathione transferases) enzymes, and their metabolism can generate intermediate and final metabolites and ROS with pro-oxidants efficacy (4-6). There are other forms of herbal extracts which may become pro-oxidant and induce oxidative stress by un-known mechanisms (3-6). These forms of herbal drugs extracts induced oxidative stress have also been demonstrated in preclinical or clinical studies, as well as some cell types (1). While cells exposed to herbal drugs extracts at short exposure times and low concentrations usually show increased cell viability, however, the powerful antioxidant extracts in high concentrations have been shown to be cytotoxic by inducing severe oxidative stress (1-6). It should be noted that herbal drugs extracts are abundant sources of polyphenols and these compounds are unstable and might be subjected to polymerization. Thus, it is essential to check that the observed biological properties are not due to polymerization of phenolic compounds (1-6). This should be verified by measuring antioxidant ability and stability of the polyphenols (2-5). Other than oxidation, pro-oxidant activity and polymerization of the herbal medicine extracts, the toxicity related to the use of crude or products of herbal medicine have also been reported (5-7). This kind of toxicity can be due to post-harvest processing and storage, adulteration, misuse of herbal medicine or contamination with toxic chemicals from cultivation (2-7). Therefore, despite various evidence supporting benefits for renal tubular cell protection, however they may also act as pro-oxidants (4-8). Care is required to prevent oxidation, induction of pro-oxidant activity and polymerization of the herbal medicine extracts, as well as the toxicity related to the use of crude or products of herbal medicine products due to post-harvest processing and storage, adulteration, misuse of medicinal plants or contamination with toxic chemicals from cultivation (4-9).

## Authors’ contributions

The authors wrote the manuscript equally.

## Conflict of interests

The authors declared no competing interests.

## Ethical considerations

Ethical issues (including plagiarism, data fabrication, double publication) have been completely observed by the author.

## Funding/Support

None declared.
